# Supplementary material for: Resolving the full spectrum of human genome variation using Linked-Reads
Source: Genome Res. 2019 Apr;29(4):635–45. doi: 10.1101/gr.234443.118 (PMC6442396; doi:10.1101/gr.234443.118)
Supplement: Supplemental Material [file supp_gr.234443.118_Supplemental_longranger-2.2.zip › longranger-2.2.tar.bz2!/lr2.2-public/lib/stan/cmdstan/stan/doc/api/html/frechet_8hpp_source.html]

Stan: src/stan/prob/distributions/univariate/continuous/frechet.hpp Source File


|  |  |
| --- | --- |
|  | Stan  2.5.0  probability, sampling & optimization |


- Main Page
- Related Pages
- Namespaces
- Classes
- Files

- File List
- File Members

All Classes Namespaces Files Functions Variables Typedefs Enumerations Enumerator Friends Macros Pages

- src
- stan
- prob
- distributions
- univariate
- continuous

frechet.hpp

Go to the documentation of this file.

1 #ifndef STAN\_\_PROB\_\_DISTRIBUTIONS\_\_FRECHET\_HPP

2 #define STAN\_\_PROB\_\_DISTRIBUTIONS\_\_FRECHET\_HPP

3

4 #include <boost/random/weibull\_distribution.hpp>

5 #include <boost/random/variate\_generator.hpp>

6

7 #include <stan/agrad/partials\_vari.hpp>

8 #include <stan/math/error\_handling.hpp>

9 #include <stan/math/functions/multiply\_log.hpp>

10 #include <stan/math/functions/value\_of.hpp>

11 #include <stan/meta/traits.hpp>

12 #include <stan/prob/constants.hpp>

13 #include <stan/prob/traits.hpp>

14

15 namespace stan {

16

17  namespace prob {

18

19  // Frechet(y|alpha,sigma) [y > 0; alpha > 0; sigma > 0]

20  // FIXME: document

21  template <bool propto,

22  typename T\_y, typename T\_shape, typename T\_scale>

23  typename return\_type<T\_y,T\_shape,T\_scale>::type

24  frechet\_log(const T\_y& y, const T\_shape& alpha, const T\_scale& sigma) {

25  static const char\* function = "stan::prob::frechet\_log(%1%)";

26

27  using stan::math::check\_positive;

28  using stan::math::check\_not\_nan;

29  using stan::math::check\_positive\_finite;

30  using stan::math::value\_of;

31  using stan::math::check\_consistent\_sizes;

32  using stan::math::multiply\_log;

33

34  // check if any vectors are zero length

35  if (!(stan::length(y)

36  && stan::length(alpha)

37  && stan::length(sigma)))

38  return 0.0;

39

40  // set up return value accumulator

41  double logp(0.0);

42  check\_positive(function, y, "Random variable", &logp);

43  check\_positive\_finite(function, alpha, "Shape parameter", &logp);

44  check\_positive\_finite(function, sigma, "Scale parameter", &logp);

45  check\_consistent\_sizes(function,

46  y,alpha,sigma,

47  "Random variable","Shape parameter",

48  "Scale parameter",

49  &logp);

50

51  // check if no variables are involved and prop-to

52  if (!include\_summand<propto,T\_y,T\_shape,T\_scale>::value)

53  return 0.0;

54

55  VectorView<const T\_y> y\_vec(y);

56  VectorView<const T\_shape> alpha\_vec(alpha);

57  VectorView<const T\_scale> sigma\_vec(sigma);

58  size\_t N = max\_size(y, alpha, sigma);

59

60  DoubleVectorView<include\_summand<propto,T\_shape>::value,

61  is\_vector<T\_shape>::value> log\_alpha(length(alpha));

62  for (size\_t i = 0; i < length(alpha); i++)

63  if (include\_summand<propto,T\_shape>::value)

64  log\_alpha[i] = log(value\_of(alpha\_vec[i]));

65

66  DoubleVectorView<include\_summand<propto,T\_y,T\_shape>::value,

67  is\_vector<T\_y>::value> log\_y(length(y));

68  for (size\_t i = 0; i < length(y); i++)

69  if (include\_summand<propto,T\_y,T\_shape>::value)

70  log\_y[i] = log(value\_of(y\_vec[i]));

71

72  DoubleVectorView<include\_summand<propto,T\_shape,T\_scale>::value,

73  is\_vector<T\_scale>::value> log\_sigma(length(sigma));

74  for (size\_t i = 0; i < length(sigma); i++)

75  if (include\_summand<propto,T\_shape,T\_scale>::value)

76  log\_sigma[i] = log(value\_of(sigma\_vec[i]));

77

78  DoubleVectorView<include\_summand<propto,T\_y,T\_shape,T\_scale>::value,

79  is\_vector<T\_y>::value> inv\_y(length(y));

80  for (size\_t i = 0; i < length(y); i++)

81  if (include\_summand<propto,T\_y,T\_shape,T\_scale>::value)

82  inv\_y[i] = 1.0 / value\_of(y\_vec[i]);

83

84  DoubleVectorView<include\_summand<propto,T\_y,T\_shape,T\_scale>::value,

85  is\_vector<T\_y>::value | is\_vector<T\_shape>::value | is\_vector<T\_scale>::value>

86  sigma\_div\_y\_pow\_alpha(N);

87  for (size\_t i = 0; i < N; i++)

88  if (include\_summand<propto,T\_y,T\_shape,T\_scale>::value) {

89  const double alpha\_dbl = value\_of(alpha\_vec[i]);

90  sigma\_div\_y\_pow\_alpha[i] = pow(value\_of(inv\_y[i]) \* value\_of(sigma\_vec[i]), alpha\_dbl);

91  }

92

93  agrad::OperandsAndPartials<T\_y,T\_shape,T\_scale> operands\_and\_partials(y,alpha,sigma);

94  for (size\_t n = 0; n < N; n++) {

95  const double alpha\_dbl = value\_of(alpha\_vec[n]);

96  if (include\_summand<propto,T\_shape>::value)

97  logp += log\_alpha[n];

98  if (include\_summand<propto,T\_y,T\_shape>::value)

99  logp -= (alpha\_dbl+1.0)\*log\_y[n];

100  if (include\_summand<propto,T\_shape,T\_scale>::value)

101  logp += alpha\_dbl\*log\_sigma[n];

102  if (include\_summand<propto,T\_y,T\_shape,T\_scale>::value)

103  logp -= sigma\_div\_y\_pow\_alpha[n];

104

105  if (!is\_constant\_struct<T\_y>::value) {

106  const double inv\_y\_dbl = value\_of(inv\_y[n]);

107  operands\_and\_partials.d\_x1[n]

108  += -(alpha\_dbl+1.0) \* inv\_y\_dbl

109  + alpha\_dbl \* sigma\_div\_y\_pow\_alpha[n] \* inv\_y\_dbl;

110  }

111  if (!is\_constant\_struct<T\_shape>::value)

112  operands\_and\_partials.d\_x2[n]

113  += 1.0/alpha\_dbl

114  + (1.0 - sigma\_div\_y\_pow\_alpha[n]) \* (log\_sigma[n] - log\_y[n]);

115  if (!is\_constant\_struct<T\_scale>::value)

116  operands\_and\_partials.d\_x3[n]

117  += alpha\_dbl / value\_of(sigma\_vec[n]) \* (1 - sigma\_div\_y\_pow\_alpha[n]);

118  }

119  return operands\_and\_partials.to\_var(logp);

120  }

121

122  template <typename T\_y, typename T\_shape, typename T\_scale>

123  inline

124  typename return\_type<T\_y,T\_shape,T\_scale>::type

125  frechet\_log(const T\_y& y, const T\_shape& alpha, const T\_scale& sigma) {

126  return frechet\_log<false>(y,alpha,sigma);

127  }

128

129  template <typename T\_y, typename T\_shape, typename T\_scale>

130  typename return\_type<T\_y,T\_shape,T\_scale>::type

131  frechet\_cdf(const T\_y& y, const T\_shape& alpha, const T\_scale& sigma) {

132

133  static const char\* function = "stan::prob::frechet\_cdf(%1%)";

134

135  using stan::math::check\_positive\_finite;

136  using stan::math::check\_positive;

137  using stan::math::check\_nonnegative;

138  using boost::math::tools::promote\_args;

139  using stan::math::value\_of;

140

141  // check if any vectors are zero length

142  if (!(stan::length(y)

143  && stan::length(alpha)

144  && stan::length(sigma)))

145  return 1.0;

146

147  double cdf(1.0);

148  check\_positive(function, y, "Random variable", &cdf);

149  check\_positive\_finite(function, alpha, "Shape parameter", &cdf);

150  check\_positive\_finite(function, sigma, "Scale parameter", &cdf);

151

152  agrad::OperandsAndPartials<T\_y, T\_shape, T\_scale>

153  operands\_and\_partials(y, alpha, sigma);

154

155  VectorView<const T\_y> y\_vec(y);

156  VectorView<const T\_scale> sigma\_vec(sigma);

157  VectorView<const T\_shape> alpha\_vec(alpha);

158  size\_t N = max\_size(y, sigma, alpha);

159  for (size\_t n = 0; n < N; n++) {

160  const double y\_dbl = value\_of(y\_vec[n]);

161  const double sigma\_dbl = value\_of(sigma\_vec[n]);

162  const double alpha\_dbl = value\_of(alpha\_vec[n]);

163  const double pow\_ = pow(sigma\_dbl / y\_dbl, alpha\_dbl);

164  const double cdf\_ = exp(-pow\_);

165

166  //cdf

167  cdf \*= cdf\_;

168

169  //gradients

170  if (!is\_constant\_struct<T\_y>::value)

171  operands\_and\_partials.d\_x1[n] += pow\_ \* alpha\_dbl / y\_dbl;

172  if (!is\_constant\_struct<T\_shape>::value)

173  operands\_and\_partials.d\_x2[n] += pow\_ \* log(y\_dbl / sigma\_dbl);

174  if (!is\_constant\_struct<T\_scale>::value)

175  operands\_and\_partials.d\_x3[n] -= pow\_ \* alpha\_dbl / sigma\_dbl;

176  }

177

178  if (!is\_constant\_struct<T\_y>::value)

179  for (size\_t n = 0; n < stan::length(y); ++n)

180  operands\_and\_partials.d\_x1[n] \*= cdf;

181  if (!is\_constant\_struct<T\_shape>::value)

182  for (size\_t n = 0; n < stan::length(alpha); ++n)

183  operands\_and\_partials.d\_x2[n] \*= cdf;

184  if (!is\_constant\_struct<T\_scale>::value)

185  for (size\_t n = 0; n < stan::length(sigma); ++n)

186  operands\_and\_partials.d\_x3[n] \*= cdf;

187

188  return operands\_and\_partials.to\_var(cdf);

189  }

190

191  template <typename T\_y, typename T\_shape, typename T\_scale>

192  typename return\_type<T\_y,T\_shape,T\_scale>::type

193  frechet\_cdf\_log(const T\_y& y, const T\_shape& alpha, const T\_scale& sigma) {

194

195  static const char\* function = "stan::prob::frechet\_cdf\_log(%1%)";

196

197  using stan::math::check\_positive\_finite;

198  using stan::math::check\_positive;

199  using stan::math::check\_nonnegative;

200  using boost::math::tools::promote\_args;

201  using stan::math::value\_of;

202

203  // check if any vectors are zero length

204  if (!(stan::length(y)

205  && stan::length(alpha)

206  && stan::length(sigma)))

207  return 0.0;

208

209  double cdf\_log(0.0);

210  check\_positive(function, y, "Random variable", &cdf\_log);

211  check\_positive\_finite(function, alpha, "Shape parameter", &cdf\_log);

212  check\_positive\_finite(function, sigma, "Scale parameter", &cdf\_log);

213

214  agrad::OperandsAndPartials<T\_y, T\_shape, T\_scale>

215  operands\_and\_partials(y, alpha, sigma);

216

217  VectorView<const T\_y> y\_vec(y);

218  VectorView<const T\_scale> sigma\_vec(sigma);

219  VectorView<const T\_shape> alpha\_vec(alpha);

220  size\_t N = max\_size(y, sigma, alpha);

221  for (size\_t n = 0; n < N; n++) {

222  const double y\_dbl = value\_of(y\_vec[n]);

223  const double sigma\_dbl = value\_of(sigma\_vec[n]);

224  const double alpha\_dbl = value\_of(alpha\_vec[n]);

225  const double pow\_ = pow(sigma\_dbl / y\_dbl, alpha\_dbl);

226

227  //cdf\_log

228  cdf\_log -= pow\_;

229

230  //gradients

231  if (!is\_constant\_struct<T\_y>::value)

232  operands\_and\_partials.d\_x1[n] += pow\_ \* alpha\_dbl / y\_dbl;

233  if (!is\_constant\_struct<T\_shape>::value)

234  operands\_and\_partials.d\_x2[n] += pow\_ \* log(y\_dbl / sigma\_dbl);

235  if (!is\_constant\_struct<T\_scale>::value)

236  operands\_and\_partials.d\_x3[n] -= pow\_ \* alpha\_dbl / sigma\_dbl;

237  }

238

239  return operands\_and\_partials.to\_var(cdf\_log);

240  }

241

242  template <typename T\_y, typename T\_shape, typename T\_scale>

243  typename return\_type<T\_y,T\_shape,T\_scale>::type

244  frechet\_ccdf\_log(const T\_y& y, const T\_shape& alpha, const T\_scale& sigma) {

245

246  static const char\* function = "stan::prob::frechet\_ccdf\_log(%1%)";

247

248  using stan::math::check\_positive\_finite;

249  using stan::math::check\_positive;

250  using stan::math::check\_nonnegative;

251  using boost::math::tools::promote\_args;

252  using stan::math::value\_of;

253

254  // check if any vectors are zero length

255  if (!(stan::length(y)

256  && stan::length(alpha)

257  && stan::length(sigma)))

258  return 0.0;

259

260  double ccdf\_log(0.0);

261  check\_positive(function, y, "Random variable", &ccdf\_log);

262  check\_positive\_finite(function, alpha, "Shape parameter", &ccdf\_log);

263  check\_positive\_finite(function, sigma, "Scale parameter", &ccdf\_log);

264

265  agrad::OperandsAndPartials<T\_y, T\_shape, T\_scale>

266  operands\_and\_partials(y, alpha, sigma);

267

268  VectorView<const T\_y> y\_vec(y);

269  VectorView<const T\_scale> sigma\_vec(sigma);

270  VectorView<const T\_shape> alpha\_vec(alpha);

271  size\_t N = max\_size(y, sigma, alpha);

272  for (size\_t n = 0; n < N; n++) {

273  const double y\_dbl = value\_of(y\_vec[n]);

274  const double sigma\_dbl = value\_of(sigma\_vec[n]);

275  const double alpha\_dbl = value\_of(alpha\_vec[n]);

276  const double pow\_ = pow(sigma\_dbl / y\_dbl, alpha\_dbl);

277  const double exp\_ = exp(-pow\_);

278

279  //ccdf\_log

280  ccdf\_log += log(1 - exp\_);

281

282  //gradients

283  const double rep\_deriv\_ = pow\_ / ( 1.0 / exp\_ - 1 );

284  if (!is\_constant\_struct<T\_y>::value)

285  operands\_and\_partials.d\_x1[n] -= alpha\_dbl / y\_dbl \* rep\_deriv\_;

286  if (!is\_constant\_struct<T\_shape>::value)

287  operands\_and\_partials.d\_x2[n] -= log(y\_dbl / sigma\_dbl) \* rep\_deriv\_;

288  if (!is\_constant\_struct<T\_scale>::value)

289  operands\_and\_partials.d\_x3[n] += alpha\_dbl / sigma\_dbl \* rep\_deriv\_;

290  }

291

292  return operands\_and\_partials.to\_var(ccdf\_log);

293  }

294

295  template <class RNG>

296  inline double

297  frechet\_rng(const double alpha,

298  const double sigma,

299  RNG& rng) {

300  using boost::variate\_generator;

301  using boost::random::weibull\_distribution;

302

303  static const char\* function = "stan::prob::frechet\_rng(%1%)";

304

305  using stan::math::check\_finite;

306  using stan::math::check\_not\_nan;

307  using stan::math::check\_positive;

308

309  check\_finite(function, alpha, "Shape parameter", (double\*)0);

310  check\_positive(function, alpha, "Shape parameter", (double\*)0);

311  check\_not\_nan(function, sigma, "Scale parameter", (double\*)0);

312  check\_positive(function, sigma, "Scale parameter", (double\*)0);

313

314  variate\_generator<RNG&, weibull\_distribution<> >

315  weibull\_rng(rng, weibull\_distribution<>(alpha, 1.0/sigma));

316  return 1.0 / weibull\_rng();

317  }

318  }

319 }

320 #endif

multiply\_log.hpp

traits.hpp

stan::prob::weibull\_rng

double weibull\_rng(const double alpha, const double sigma, RNG &rng)

**Definition:** weibull.hpp:304

stan::prob::frechet\_ccdf\_log

return\_type< T\_y, T\_shape, T\_scale >::type frechet\_ccdf\_log(const T\_y &y, const T\_shape &alpha, const T\_scale &sigma)

**Definition:** frechet.hpp:244

stan::agrad::OperandsAndPartials::to\_var

T\_return\_type to\_var(double logp)

**Definition:** partials\_vari.hpp:138

stan::math::check\_positive\_finite

bool check\_positive\_finite(const char \*function, const T\_y &y, const char \*name, T\_result \*result)

**Definition:** check\_positive\_finite.hpp:12

stan::agrad::pow

fvar< T > pow(const fvar< T > &x1, const fvar< T > &x2)

**Definition:** pow.hpp:17

error\_handling.hpp

stan::math::multiply\_log

boost::math::tools::promote\_args< T\_a, T\_b >::type multiply\_log(const T\_a a, const T\_b b)

Calculated the value of the first argument times log of the second argument while behaving properly w...

**Definition:** multiply\_log.hpp:51

stan::length

size\_t length(const T &)

**Definition:** traits.hpp:159

stan::DoubleVectorView

DoubleVectorView allocates double values to be used as intermediate values.

**Definition:** traits.hpp:358

stan::math::check\_finite

bool check\_finite(const char \*function, const T\_y &y, const char \*name, T\_result \*result)

Checks if the variable y is finite.

**Definition:** check\_finite.hpp:52

partials\_vari.hpp

stan::agrad::value\_of

T value\_of(const fvar< T > &v)

Return the value of the specified variable.

**Definition:** value\_of.hpp:16

constants.hpp

stan::is\_vector

**Definition:** traits.hpp:83

stan::agrad::OperandsAndPartials

A variable implementation that stores operands and derivatives with respect to the variable...

**Definition:** partials\_vari.hpp:76

traits.hpp

stan::return\_type::type

boost::math::tools::promote\_args< typename scalar\_type< T1 >::type, typename scalar\_type< T2 >::type, typename scalar\_type< T3 >::type, typename scalar\_type< T4 >::type, typename scalar\_type< T5 >::type, typename scalar\_type< T6 >::type >::type type

**Definition:** traits.hpp:406

stan::is\_constant\_struct

Metaprogram to determine if a type has a base scalar type that can be assigned to type double...

**Definition:** traits.hpp:57

stan::math::value\_of

double value\_of(const T x)

Return the value of the specified scalar argument converted to a double value.

**Definition:** value\_of.hpp:24

stan::prob::frechet\_log

return\_type< T\_y, T\_shape, T\_scale >::type frechet\_log(const T\_y &y, const T\_shape &alpha, const T\_scale &sigma)

**Definition:** frechet.hpp:24

stan::prob::include\_summand

Template metaprogram to calculate whether a summand needs to be included in a proportional (log) prob...

**Definition:** traits.hpp:35

stan::agrad::OperandsAndPartials::d\_x2

VectorView< double \*, is\_vector< T2 >::value, is\_constant\_struct< T2 >::value > d\_x2

**Definition:** partials\_vari.hpp:83

stan::math::check\_nonnegative

bool check\_nonnegative(const char \*function, const T\_y &y, const char \*name, T\_result \*result)

**Definition:** check\_nonnegative.hpp:52

stan::math::check\_consistent\_sizes

bool check\_consistent\_sizes(const char \*function, const T1 &x1, const T2 &x2, const char \*name1, const char \*name2, T\_result \*result)

**Definition:** check\_consistent\_sizes.hpp:13

stan::max\_size

size\_t max\_size(const T1 &x1, const T2 &x2)

**Definition:** traits.hpp:191

stan::math::check\_positive

bool check\_positive(const char \*function, const T\_y &y, const char \*name, T\_result \*result)

**Definition:** check\_positive.hpp:54

stan::prob::frechet\_cdf

return\_type< T\_y, T\_shape, T\_scale >::type frechet\_cdf(const T\_y &y, const T\_shape &alpha, const T\_scale &sigma)

**Definition:** frechet.hpp:131

stan::math::check\_not\_nan

bool check\_not\_nan(const char \*function, const T\_y &y, const char \*name, T\_result \*result)

Checks if the variable y is nan.

**Definition:** check\_not\_nan.hpp:56

stan::prob::frechet\_rng

double frechet\_rng(const double alpha, const double sigma, RNG &rng)

**Definition:** frechet.hpp:297

value\_of.hpp

stan::agrad::OperandsAndPartials::d\_x1

VectorView< double \*, is\_vector< T1 >::value, is\_constant\_struct< T1 >::value > d\_x1

**Definition:** partials\_vari.hpp:82

stan::agrad::OperandsAndPartials::d\_x3

VectorView< double \*, is\_vector< T3 >::value, is\_constant\_struct< T3 >::value > d\_x3

**Definition:** partials\_vari.hpp:84

stan::agrad::log

fvar< T > log(const fvar< T > &x)

**Definition:** log.hpp:15

stan::VectorView

VectorView is a template metaprogram that takes its argument and allows it to be used like a vector...

**Definition:** traits.hpp:275

stan::agrad::exp

fvar< T > exp(const fvar< T > &x)

**Definition:** exp.hpp:16

stan::prob::frechet\_cdf\_log

return\_type< T\_y, T\_shape, T\_scale >::type frechet\_cdf\_log(const T\_y &y, const T\_shape &alpha, const T\_scale &sigma)

**Definition:** frechet.hpp:193


---

[ Stan Home Page ]

*© 2011–2014,
Stan Development Team.*
